# Fatty acid, physicochemical composition and sensory attributes of meat from lambs fed diets containing licuri cake

**DOI:** 10.1371/journal.pone.0206863

**Published:** 2018-11-16

**Authors:** Jonival Barreto Costa, Ronaldo Lopes Oliveira, Thadeu Mariniello Silva, Analívia Martins Barbosa, Máikal Souza Borja, Caius Barcellos de Pellegrini, Vinicius da Silva Oliveira, Rebeca Dantas Xavier Ribeiro, Leilson Rocha Bezerra

**Affiliations:** 1 Department of Veterinary Medicine and Animal Science, Federal University of Bahia, Salvador, Bahia, Brasil; 2 Department of Animal Science, Federal University of Piaui, Rodovia Bom Jesus-Viana, Bom Jesus, Piaui, Brazil; University of Illinois, UNITED STATES

## Abstract

The aim of this study was to determine the effect of feeding licuri cake to lambs on the sensory characteristics, physicochemical characteristics and fatty acid (FA) profile of meat from lambs. Forty-four crossbred Santa Ines lambs (21.2 ± 2.70 kg body weight; 6 months old) were housed in individual pens and fed 4 experimental diets, containing 0, 8, 16 or 24% licuri cake (DM basis). The averages concentrations of ash (11.4), pH (5.82), lightness (38.1), cooking loss (26.8) or shear-force resistance (2.48) of lamb meat were not affected by the licuri cake diets. However, there was a linear decrease (*P* < 0.01) of redness and chroma indexes, lipid and protein contents, whereas the moisture content of the meat (*P* < 0.001) increased linearly due to the inclusion of licuri cake in lambs’ diets. The licuri cake inclusion in the lambs feed linearly increased (*P* < 0.05) the fatty acids concentrations of C12:0, C17:0, C20:0, C20:1, C18:3, C20:3, C20:4 and ΣPUFA/ΣMUFA ratio, Σ*ω–3* and atherogenicity index (AI). However, C18:1 *cis*, C20:2, C20:5, ΣMUFA, ΣMUFA/ΣSFA and Σ*ω–6*:Σ*ω–3* ratios in the *longissimus lumborum* of lambs linearly decreased by licuri cake inclusion. There was a quadratic increase (*P* < 0.05) on C14:0 (maximum point 4.94 g/100 g FAME to 14.5% licuri inclusion), C16:1 (maximum point 8.59 g/100 g FAME to 10.7% licuri inclusion) and enzymatic activities of Δ^9^-desaturase C16 (maximum point 27.5 g/100 g FAME to 10.6% licuri inclusion) in the *longissimus lumborum* of lambs fed due to increased concentrations of licuri cake. However, there was a quadratic decrease (*P* = 0.04) in ΣPUFA/ΣSFA ratio with minimum concentration of 0.63 g/100 g FAME to 11.1% inclusion. The inclusion of licuri cake in the lambs diet did not change (*P* > 0.05) the concentrations of SFA C10:0, C15:0, C16:0, C18:0, C14:1, MUFA C18:1 trans, PUFA C18:2 *cis*, CLA, total sum of ΣSFA and ΣPUFA, desirable fatty acids (DFA), hypocholesterolemic:hypercholesterolemic index, and elongase and Δ^9^-desaturase C18 enzymes. Licuri cake in the lamb diet improved (*P* < 0.05) meat aroma, flavor and overall acceptance by consumers. Licuri cake inclusion in the diet of lambs improves sensory attributes of meat and the meat fatty acid profile becomes nutritionally healthier for the human diet because do not affect major FA of meat; however, the growth performance of finishing lambs is reduced.

## Introduction

Current research seeks feeds that can replace traditional ingredients, such as corn and soybeans, with the goal of reducing costs without affecting productivity [1–5]. The byproducts of biodiesel production, such as licuri cake, represent important alternative feeds for the production of high-quality lamb meat [4,6,7]. Vegetable cakes have been used in lambs’ diets because they have high amount of unsaturated fatty acids (UFAs); especially oleic, linoleic and linolenic acids [4,6]; which increases deposition efficiency and the quality of deposited fat, important components in the meat industry.

Furthermore, it is possible to increase lamb meat quality by decreasing concentrations of saturated fatty acids (SFAs) and increasing mono- and polyunsaturated fatty acids (MUFAs and PUFAs, respectively) content in lipid profile, thus increasing the formation of compounds that are beneficial to human health, such as conjugated linoleic acid (CLA) and the isomer groups *ω–3* and *ω–6*, as well as the *ω–3*/*ω–6* ratio [4,6,8], which can decrease the risk of appearance of atherogenic plaque on arteries of meat consumers and may contribute to the prevention of cardiovascular disease [9].

Licuri (*Syagrus coronata*) cake, a biodiesel byproduct of licuri oil generated from mechanical pressing during licuri fruit processing, contains approximately 24% crude protein (CP), 10% ether extract (EE), 52% neutral detergent fiber (NDF) and 35% g/kg acid detergent fiber (ADF) on a dry matter (DM) basis [7,10,11]. It can be used as an ingredient in the feed given to confined lambs and can increase the CP content of the meat without affecting carcass and non-carcass components [12]. Thus, we hypothesized that including licuri cake in a lamb’s diet would increase meat quality and beneficially modify its fatty acid (FA) profile, thereby increasing product acceptance by the consumer. The objective was to determine the effects of licuri cake inclusion on the physicochemical, FA profile and sensory characteristics of lamb meat.

## Materials and methods

This study was carried out in strict accordance with the recommendations of the animal care guidelines of the National Council for the Control of Animal Experimentation, Brazil. The protocol was approved by the Committee on the Ethics of Animal Experiments of the University of Bahia, Bahia State, Brazil (Permit Number: 02–2014).

### Animals, treatments, diets and chemical analyses

Forty-four crossbred non-castrated Santa Ines lambs, with an average age of six months and an initial weight of 21.2 ± 2.70 kg, were distributed in a completely randomized design across 4 treatments and 11 replicates. The treatments were different concentrations of licuri cake included in the feed: 0 (control group), 8%, 16% and 24% of total DM (Table 1).

**Table 1:** Chemical composition of experimental diets with included licuri cake and the proportions of ingredients fed to crossbred lambs.

Item	Licuri cake (%total DM)			
	0	8	16	24
Ingredients proportion (% total DM)				
Ground corn	40.3	35.6	30.8	26.0
Soybean meal	17.2	14.0	10.7	7.50
Licuri cake	0.00	8.00	16.0	24.0
Mineral mixture^a^	1.50	1.50	1.50	1.50
Urea + ammonium sulfate^b^	1.00	1.00	1.00	1.00
Tifton-85 hay	40.0	40.0	40.0	40.0
Chemical composition of diets (% total DM)				
Dry matter (% as fed)	89.8	89.7	89.6	89.5
Ash	5.49	5.70	5.92	6.13
Crude protein	16.1	16.1	16.1	16.1
Ether extract	2.60	3.43	4.27	5.10
Neutral detergent fiber^c^	38.3	41.0	43.7	46.4
Acid detergent fiber	19.3	21.1	22.9	24.8
Neutral detergent insoluble protein	25.9	25.7	25.4	25.1
Acid detergent insoluble protein	7.87	7.48	7.10	6.71
Acid detergent lignin	3.12	3.93	4.74	5.55
Cellulose	16.1	17.2	18.2	19.2
Hemicellulose	19.0	19.9	20.7	21.6
Non-fibrous carbohydrates	37.6	33.8	30.0	26.3
Dry matter intake (g/d)	1195	1102	877	658
Average daily gain (kg/day)	0.20	0.21	0.16	0.11
Slaughter weight (kg)	37.0	36.1	33.4	30.6

^a^Mineral mixture—levels of guarantee per kg: calcium 120 g, phosphorus 87 g, sodium 147 g, sulfur 18 g, copper 590 mg, cobalt 40 mg, chromium 20 mg, iron 1,800 mg, iodine 80 mg, manganese 1,300 mg, selenium 15 mg, zinc 3,800 mg, molybdenum 300 mg, and fluoride at a maximum of 870 mg.

^b^Mixture of urea and ammonium sulfate, at a ratio of 9:1 parts.

^c^Corrected for ash and protein contents.

The experiment included 18 d of adaptation followed by 70 d of sample collection, for a total of 88 d. The animals were housed in a covered shed in individual 1.6-m^2^ pens with ad libitum access to water and feed.

The total mixed ration (TMR) was a mixture of roughage, Tifton-85 (*Cynodon* sp.) hay, and concentrate made from soybean meal, ground corn, a mineral mixture, and licuri cake, with a forage: concentrate ratio of 400:600 g/kg (Table 1). A large supply of Tifton-85 grass hay was ground into particles approximately 5 cm in length. The concentrate was homogenized in an automatic feed mixer and later blended with the roughage. The licuri cake was ground to break up lumps and to facilitate homogenization with the other ingredients in the concentrate.

The animals were fed twice daily at 09:00 and 16:30, and feed and refusals were weighed daily to monitor feed intake; feeding was adjusted to ensure that refusals were between 5 and 10% of total DM. Isonitrogenous diets were formulated based on the recommendations of the National Research Council [13] to meet the nutritional requirements for crossbred lambs and allow for estimated weight gains of 200 g/day. Nutrient intake was estimated from the difference between the total amount of each nutrient contained in the feed offered and the amount in the refusals.

Triplicate samples of ingredients, diets and refusals were dried at 55°C for 72 h in a forced-air oven, ground with a Willey mill (Tecnal, Piracicaba City, São Paulo State, Brazil) with a 1-mm sieve, and stored in airtight plastic containers (ASS, Ribeirão Preto City, São Paulo, Brazil). The samples were stored in plastic jars with lids (ASS, Ribeirão Preto, São Paulo, Brazil), labeled and subjected to further laboratory analysis to measure the contents of DM (method 967.03), ash (method 942.05), CP (method 981.10), and EE (method 920.29) according to the AOAC [14].

The NDF content was determined as described by Van Soest et al. [15], with modifications as proposed in the ANKOM fiber analyzer manual (ANKOM Technology Corporation, Macedon, NY, USA); heat-stable alpha-amylase was not used. The ADF contents were determined as described by Robertson and Van Soest [16]. NDF residue was incinerated in an oven at 600°C for 4 h to determine the ash content, and the protein correction was calculated by subtracting the neutral detergent insoluble protein (NDIP) content. NDF was corrected for the ash and protein contents. The acid detergent lignin (ADL) content was determined according to method 973.18 [17], in which ADF residue was treated with 72% sulfuric acid. Non-fiber carbohydrates (NFCs) were measured according to Mertens [18] and calculated based on the equation NFC = 100 –NDF–CP–EE–ash. The NDIP and acid detergent insoluble protein (ADIP) values were obtained according to Licitra et al. [19].

### Slaughter

Lambs were weighed at the beginning of the experiment (initial weight) and at the end of the experiment (final weight) to determine average daily gain (ADG; Table 1). Lambs were subjected to 16 h of fasting and then weighed again to determine slaughter weight. After being weighed, the lambs were slaughtered using a captive bolt gun (Dal Pino, São Paulo, Brazil) to stun them and promote electronarcosis (minimum current of 1.25 amperes), followed by bleeding, skinning and gutting according to the rules established in the Regulations of Brazilian Industrial and Sanitary Inspection of Animal Products.

The carcasses were washed, split longitudinally into two parts with an electric saw (Ki Junta, São Paulo, Brazil), weighed and chilled in a cold room at 4°C for 24 h. After that, samples from the *longissimus lumborum* muscles were desiccated, vacuum packed, frozen and stored at -20°C for later use in evaluating meat quality on the basis of physicochemical, FA profile and sensory characteristics.

### Physicochemical composition

The mean pH was calculated as the average of the pH values at 45 min (initial) and 24 h after slaughter (final) in the *longissimus lumborum* muscle, which were measured using a Mettler M1120x pH meter (Mettler, Toledo International Inc., Columbus, USA) according to AOAC [14] procedures.

The cooking loss (CL) was determined according to the American Meat Science Association [20] using quadruplicate samples without visible connective tissue that had been thawed at 10°C 12 h. Steaks were weighed before cooking. The cooking was realized in a preheated grill (George Foreman Jumbo Grill GBZ6BW, Rio de Janeiro, Brazil) at 170°C. The digital thermometer (Salcasterm 200, São Paulo, Brazil) was used to monitor the internal temperature of each steak until the center reached 71°C. Each steak was brought to room temperature, removed from the grill after temperature stabilization, and weighed again. Cooking loss was calculated by the difference between the initial and final weights of each steak, with the value expressed as a percentage.

Cubes (triplicate) of at least 1.00 cm in width were cut from the muscles longitudinal to the fibers to be evaluated for Warner-Bratzler shear force (WBSF). Instrumental texture analysis was performed on a TAXT2 texturometer (Stable Micro Systems Ltd., Vienna Court, UK) at 200 mm/min using standard shearing blades (1.016 mm thick with a 3.05-mm blade) according to Shackelford et al. [21].

Meat color was determined after thawing in a refrigerator at 4°C for 12 h and exposure to the atmosphere for 30 min, based on the myoglobin oxygenation that the main molecule that defines the color of the meat [22]. Coordinates L*, a* and b* were measured at three different points on the muscle sample, and triplicate measurements were averaged for each coordinate per animal. These measurements were performed using a Minolta CR-10 colorimeter (Konica Minolta, Osaka, Japan) that was previously calibrated with the CIELAB system using a blank tile with illuminant D65 and 10° as the standard observation point. L*represents lightness (L* = 0 black, 100 white); a* (redness) ranges from green (−) to red (+); and b*(yellowness) ranges from blue (−) to yellow (+). Measurements were made at a 2° viewing angle using illuminant C. Color saturation (chroma, C*) was calculated as (a*^2^ + b*^2^)^1/2^, according to Boccard et al. [23]. Three readings were performed on each muscle sample to obtain a mean value for each animal.

Determination of moisture, ash and CP content followed AOAC (2000) recommendations. The lipid extract used to determine the FA profiles of the samples was obtained using a technique described by Bligh and Dyer [24], with adaptations, and using a 2:1 chloroform: methanol solution as the solvent. After extraction, lipids were esterified and methylated according to the method described by Hartman and Lago [25] to determine the FA profiles of the meat and diets.

### Fatty acid profile

The quantification and determination of FAs were performed in the ingredients of diets (Table 2) and meat in triplicate using a gas chromatograph mass spectrometer Shimadzu (GCMS-QP2010 SE), RT-x Wax Polietileno Glicol column (30 m in length, 0.25-mm internal diameter and 0.25-μm film thickness).

**Table 2:** Fatty acids composition (g/100 g FAME) in the ingredients of the experimental diets.

Fatty Acids (g/100 g FAME)^A^	Ingredients			
	Ground corn	Soybean meal	Licuri cake	Tifton-85 hay
C12:0	0.01	0.02	2.61	3.78
C14:0	0.08	0.16	8.68	2.02
C16:0	15.3	16.8	17.6	15.5
C18:0	2.05	3.56	11.3	8.95
C18:1 *cis*	33.6	22.6	21.5	22.4
C18:1 *trans*	0.03	0.05	0.01	0.08
C18:2 *n−6*	47.2	51.8	35.3	42.4
C18:3 *n−3*	1.02	4.49	1.62	3.62
C20:0	0.43	0.18	1.23	0.14
C22:0	0.17	0.23	0.05	0.78
C24:0	0.15	0.14	0.10	0.38

The column oven temperature was as follows: the initial temperature of 100°C was increased at 5°C/min to 190°C and at 5°C/min to 220°C and then maintained for 5 min at a rate 2°C/min. Finally, it was raised to 240°C at a rate of 5°C/min and maintained for 5 min.

A carrier gas, helium (He), was used at a flow rate of 1 mL/min, and the split ratio was 1:30. The injector temperature and detector used were 250°C. The methyl esters quantification of fatty acids was based on the area normalization [26], the samples and the standard were injected into the chromatograph together with an internal standard (heptadecanoic acid, SIGMA-ALDRICH, St. United States). The internal standard concentration used was 25.5 mg/ L. The FAMEs were identified by a comparison of the FAME retention times with those of authentic standards (FAME Mix, C4-C24, SIGMA-ALDRICH, St. Louis, USA). A response factor was generated for each fatty acid based on the sample in the standard, and the response factor of each fatty acid was obtained to quantify the fatty acid methyl esters [27]. The results were quantified by normalizing the areas of the methyl esters and are expressed as g 100/g fatty acids methyl esters (FAME) (Table 2).

The values of the SFAs, UFAs, MUFAs, PUFAs, omega 6 (*n–6*), and omega 3 (*n–3*) were summed from the profiles of identified FAs in *longissimus lumborum* muscle samples, and the PUFA/SFA, *n–6*/*n–3*, and SFA/UFA ratios were determined. The atherogenicity index (AI) was determined according to Ulbricht and Southgate [9] using the equation AI = [(C12:0 + (4 × C14:0) + C16:0)]/(ΣMUFA + Σ*n–6* + Σ*n–3*). The hypocholesterolemic to hypercholesterolemic fatty acid ratio was determined by the formula h:H = (C18:1*cis-*9 + C18:2*n-6* + 20:4*n-6*+C18:3*n-3*+ C20:5*n-3*+ C22:5*n-3* + C22:6*n-3*)/(C14:0+C16:0), according to Arruda *et al*. [28], and the desirable fatty acids = (MUFA+PUFA+C18:0), according to Rhee [29]. The activities of Δ9-desaturase C16 (D9C16), Δ9-desaturase C18 (D9C18) and elongase were estimated according to De Smet *et al*. [30] with the following equations: D9C16 = [C16:1/ (C16:0 + C16:1)] × 100, D9C18 = [(C18:1 cis–9) / (C18:0 + C18:1 cis–9)] × 100 and elongase = [(C18:0+ C18:1 cis–9)/(C16:0 + C16:1 + C18:0 + C18:1 cis–9)] × 100.

### Sensorial analyses

This research was also submitted to and approved by the Brazil Platform regarding its ethical and methodological aspects according to the guidelines established in Resolution 466/2012 and complementary to those of the National Health Council, which were approved by the Research Ethics Committee of the Federal University of Piaui. Before participating in the sensorial meat attribute tests, the participants signed informed consent forms. The responsible researcher signed a terms of responsibility form, ensuring that the participants’ identifications were kept confidential.

The sensory characteristics of *longissimus lumborum* samples were evaluated by 70 untrained consumer panelists [31]. We have added some information in this methodology: "All panelists were animal science department members, 35 women and 35 men, comprising an age group between 19 and 55 years old. Three-cm^3^ cubes were cut from the loin and grilled on an electric grill (George Foreman Grill Jumbo GBZ6BW, Rio de Janeiro, Brazil) for eight min (four min on each side) until the temperature of the geometric center reached 71°C. After heating, the meat samples (in duplicate) from lambs fed different concentrations of licuri cake (0.00, 80.0, 160 and 240 g/kg total DM), the samples were wrapped in aluminum foil. No salt or condiments were added. Water and cream cracker-type biscuits accompanied the meat samples to remove the aftertaste between tastings.

Tests have performed between 09:00 and 12:00 am. In each round, 5 people entered, and they stayed in individual booths for about 10 minutes to taste and evaluate of the meats. The sensory attributes have recorded using a hedonic scale of nine points (1, very much disliked; 2, disliked; 3, moderately disliked; 4, slightly disliked; 5, indifferent; 6, slightly liked; 7, moderately liked; 8, liked; and 9, very much liked). Consumer panelists evaluated the following attributes: appearance, aroma, flavor, tenderness, juiciness, overall acceptability and preference.

### Statistical analyses

The experimental design was completely randomized, with 4 treatments and 11 replications. The statistical model used was as follows: *Y*_*ij*_ *= μ + si +e*_*ij*_, where Y_ij_ = observed value; μ = overall mean; si = effect of licuri cake concentration in the diet; and e_ij_ = effect of experimental error. Polynomial contrasts were used to determine the linear and quadratic effects of the different treatment levels. The initial weight of the animals was used in the statistical model as a covariate when significant. The regression equation was adjusted when significant differences (*P* < 0.05) were detected, and tests were performed using PROC REG in SAS version 9.2 [32].

Using a completely randomized design with 70 evaluators, the scores for appearance, aroma, flavor, tenderness, juiciness and overall acceptability were analyzed to determine whether the assumptions of an analysis of variance were satisfied; none of these parameters showed a normal distribution of residuals. Therefore, we used the nonparametric Friedman test, which considered the assessor as a block, to compare the effects of licuri cake treatment at a 5% level of significance. When the Friedman test was significant, the least significant difference (LSD) was calculated to determine whether the average values differed at the *P* < 0.05 level of significance.

## Results

### Physicochemical composition

The inclusion of different concentration of licuri cake in the diets of lambs resulted in a linear increase in the moisture (*P* < 0.001) content, linear decreases in the lipid (*P* < 0.001) and protein (*P* < 0.001) contents and a quadratic decrease (*P* = 0.044) in the lipid content of lamb meat (Table 3).

**Table 3:** Physicochemical composition of *longissimus lumborum* muscle samples from crossbred finishing lambs on a feedlot and fed licuri cake.

Variable	Licuri cake (% total DM)				SEM^a^	*P–value*^b^	
	0	8	16	24		Linear	Quadratic
pH	5.85	5.68	5.92	5.82	0.12	0.758	0.685
Lightness (L*)	37.3	38.5	37.8	38.9	0.57	0.234	0.524
Redness (a*)	19.2	18.9	19.2	16.9	0.86	<0.001	0.942
Yellowness (b*)	6.40	7.07	6.50	7.77	0.53	0.091	0.509
Chroma (C*)	20.2	20.2	20.3	18.6	0.72	<0.001	0.584
Cooking loss (CL), %	26.6	25.9	25.5	29.0	1.58	0.195	0.085
Warner-Bratzler shear force, kgf/cm^2^	2.44	2.48	2.47	2.54	0.02	0.724	0.923
Moisture, g/kg	758	755	769	785	0.39	<0.001	0.117
Lipid, g/kg	23.8	26.6	21.4	14.7	0.23	0.002	0.044
Protein, g/kg	207	207	197	188	0.37	<0.001	0.242
Ash, g/kg	11.1	11.0	11.9	11.6	0.01	0.243	0.364

^a^SEM = Standard error of the mean.

^b^Significant at *P*<0.05

*Longissimus lumborum* muscle samples from lambs fed diets with different licuri cake concentrations did not show differences in ash content (*P* = 0.243; mean = 11.4), pH (*P* = 0.758; mean = 5.82), L* (*P* = 0.234; mean = 38.1), CL (*P* = 0.195; mean = 26.8) or WBSF (*P* = 0.724; mean = 2.48). Redness (a*; *P* < 0.001) and chroma (C*; *P* < 0.001) decreased linearly, whereas yellowness (b*; *P* = 0.091) increased linearly with higher concentrations of licuri cake (Table 3).

### Fatty acid profile

The inclusion of licuri cake in the lambs feed linearly increased (*P* < 0.05) the fatty acids concentrations (in units of g/100 g FAME) of lauric acid (C12:0), heptadecanoic acid (C17:0), arachidic acid (C20:0), gondoic acid (C20:1), α-linolenic acid (C18:3), eicosatrienoic acid (C20:3), arachidonic acid (C20:4) and the ΣPUFA/ΣMUFA ratio, the Σ*ω–3* and the atherogenecity index (AI). However, oleic acid (C18:1 *cis*), C20:2, eicosapentaenoic-EPA (C20:5), ΣMUFA, ΣMUFA/ΣSFA and Σ*ω–6*: Σ*ω–3* ratios in the *longissimus lumborum* of lambs linearly decreased by licuri cake inclusion (Table 4).

**Table 4:** Fatty acid composition (g/100 g FAME) in *longissimus lumborum* muscle samples of crossbred finishing lambs on a feedlot and fed licuri cake.

Fatty acids (g/100 g FAME)	Licuri cake (% total DM)				SEM^a^	P-value ^b^	
	0	8	16	24		Linear	Quadratic
Saturated (SFA)							
C10:0	0.35	0.43	0.33	0.32	0.03	0.15	0.12
C12:0	0.13	0.26	0.33	0.51	0.04	<0.001	0.65
C14:0	3.04	5.01	4.50	4.32	0.34	0.04	<0.001
C15:0	0.57	0.75	0.65	0.65	0.05	0.60	0.09
C16:0	22.5	21.1	24.3	21.2	0.97	0.88	0.40
C17:0	1.07	1.20	1.21	1.81	0.16	<0.001	0.16
C18:0	5.00	6.04	4.96	5.20	0.41	0.80	0.38
C20:0	0.08	0.05	0.08	0.18	0.02	<0.001	0.01
Monounsaturated (MUFA)						
C14:1	0.74	0.72	0.66	0.55	0.10	0.24	0.71
C15:1	0.27	0.35	0.32	0.34	0.03	0.18	0.26
C16:1	7.03	8.91	7.85	6.56	0.43	0.22	<0.001
C18:1-*cis*	33.9	32.3	29.7	30.1	1.34	0.03	0.45
C18:1-*trans*	0.41	0.40	0.45	0.44	0.04	0.49	0.86
C20:1	0.20	0.23	0.19	0.31	0.03	0.05	0.22
Polyunsaturated (PUFA)						
C18:2- *cis* 9,12	13.7	12.1	12.8	15.1	1.14	0.38	0.11
CLA^c^	3.20	2.24	2.67	2.55	0.70	0.63	0.56
C18:3	0.75	0.97	1.28	1.93	0.23	<0.001	0.41
C20:2	0.98	0.48	0.51	0.55	0.13	0.04	0.05
C20:3	0.49	0.77	1.37	1.57	0.13	<0.001	0.81
C20:4	4.28	4.89	5.34	5.50	0.41	0.03	0.58
C20:5	1.30	0.78	0.53	0.39	0.23	0.03	0.51
Sums and ratios							
∑SFA	32.7	34.8	36.3	34.2	1.11	0.27	0.08
∑MUFA	42.5	42.9	39.1	38.3	1.28	0.01	0.65
∑PUFA	24.7	22.3	24.5	27.5	1.44	0.11	0.07
MUFA:SFA	1.33	1.24	1.10	1.13	0.06	0.01	0.31
PUFA:SFA	0.78	0.64	0.70	0.82	0.06	0.48	0.04
PUFA:MUFA	0.59	0.53	0.64	0.74	0.05	0.03	0.15
∑*ω–3*	1.25	1.74	2.65	3.50	0.31	<0.001	0.61
∑*ω–6*	15.0	12.9	13.3	15.4	1.18	0.77	0.09
∑*ω–6*:∑*ω–3*	13.68	8.22	5.94	5.21	1.17	<0.001	0.06
AI^d^ index	0.60	0.72	0.79	0.70	0.05	0.11	0.03
h/H^e^ index	0.58	0.51	0.52	0.70	0.06	0.21	0.07
DFA^f^	72.3	71.2	68.6	71.0	1.08	0.22	0.13
Desaturase index							
Δ9-desaturase C16^g^	23.9	29.7	24.4	23.7	1.27	0.31	0.01
Δ9-desaturase C18^h^	86.9	84.0	85.6	85.0	1.25	0.49	0.38
Elongase activity^i^	56.9	55.9	52.1	55.9	1.38	0.30	0.09

^a^SEM = Standard error of the mean.

^b^Significant at *P* < 0.05

^c^CLA = conjugated linoleic acid (C18:2*–cis9trans11)*

^d^Atherogenicity index = [(C12:0 + (4 × C14:0) + C16:0)]/ (ΣMUFA +Σ*ω–3* + Σ*ω–6*)

^e^Hypocholesterolemic and hypercholesterolemic fatty acids ratio = (C18:1*cis ω–9* + C18:2*ω–6*) / (C14:0 + 16:0)

^f^Desirable fatty acids = (MUFA+PUFA+C18:0)

^g^Δ 9-desaturase C16 = [C16:1/ (C16:0 + C16:1)] × 100)

^h^Δ 9-desaturase C18 = [(C18:1 *cis* ω–9) / (C18:0 + C18:1*cis ω–9*)] × 100

^i^Elongase = ([(C18:0+ C18:1*cis* ω–9) / (C16:0 + C16:1 + C18:0 + C18:1 *cis* ω–9)] × 100)

There was a quadratic increase (*P* < 0.05) on C14:0 (maximum point 4.94 g/100 g FAME to 14.5% licuri inclusion), C16:1 (maximum point 8.59 g/100 g FAME to 10.7% licuri inclusion) and enzymatic activities of Δ^9^-desaturase C16 (maximum point 27.5 g/100 g FAME to 10.6% licuri inclusion) in the *longissimus lumborum* of lambs fed due to increased concentrations of licuri cake. However, there was a quadratic decrease (*P* = 0.04) in ΣPUFA/ΣSFA ratio with minimum concentration of 0.63 g/100 g FAME to 11.1% inclusion.

The inclusion of licuri cake in the lambs feed did not change (*P* > 0.05) the concentrations of capric acid (C10:0; mean = 0.36), pentadecylic acid (C15:0; mean = 0.66), palmitic acid (C16:0; mean = 22.3), stearic acid (C18:0; mean = 5.30), myristoleic acid (C14:1; mean = 0.67), C18:1 trans (mean = 0.43), linoleic acid (C18:2- *cis* 9,12; mean = 13.4), CLA (mean = 2.67), ΣSFA (mean = 34.5) and ΣPUFA (mean = 24.8), desirable fatty acids (DFA; mean = 70.8), hypocholesterolemic:hypercholesterolemic index (h/H index; mean = 0.58), enzymatic activities of elongase (mean = 55.2) and Δ^9^-desaturase C18 (mean = 85.4) in the *longissimus lumborum* of lambs.

### Sensorial analysis

The sensory characteristics of lamb meat (appearance, tenderness and juiciness) were not affected by including licuri cake in the diets (Table 5). However, the aroma (P = 0.009), flavor (P = 0.003), and overall acceptance (P = 0.044) of the meat were significantly affected by the inclusion of licuri cake.

**Table 5:** Average points (hedonic scale) for the effect of each treatment on the sensory characteristics of the longissimus lumborum of crossbred finishing lambs on a feedlot and fed licuri cake.

^a^Sensory Characteristic	Licuri cake (% total DM)				FT^a^	*P–*value^b^
	0	8	16	24		
Appearance	6.74	6.86	6.83	6.98	4.34	0.227
Aroma	6.93b	6.48b	6.83b	7.38a	11.5	0.009
Flavor	6.17b	6.24b	6.71a	7.12a	13.8	0.003
Tenderness	6.57	7.14	6.95	6.81	2.28	0.517
Juiciness	6.52	6.29	6.60	6.38	2.74	0.434
Overall acceptance	6.50b	6.48b	6.98ab	7.17a	8.15	0.044

^a^Nonparametric Friedman Test.

^b^Different letters in the same line indicate significant differences among the treatments.

The panelists identified the best aroma (*P* = 0.003) in the meat of lambs fed a diet supplemented with licuri cake at 24% of total DM; the other treatments were not significantly different from one another. Additionally, the flavors of meat from lambs fed diets supplemented with 16% and 24% licuri cake were not significantly different (*P* = 0.003), but they were significantly different from the treatments supplemented with 0% and 8% licuri cake, which were similar to one another.

The treatment supplemented with licuri cake at 24% of total DM differed in overall acceptance from the treatments supplemented with 0% and 8% licuri cake and, together with the 16% treatment group, presented the best overall acceptance (*P* = 0.044).

## Discussion

### Physicochemical composition

The linear increase in the moisture content of lamb meat was explained by the linear decrease in the lipid content as the licuri cake concentration in the diet increased. The decrease in the deposition of lipids in the meat most likely occurred due to the decrease in dry matter intake (DMI) and consequently decreased ADG [7] in the animals that received diets with the highest licuri cake concentrations. Lipid concentrations are negatively correlated with moisture content [33], and the meat of lambs fed licuri cake at 24% of total DM had the greatest moisture content because the meat lipid amount was low. Lipid is an important meat component, conferring the desirable characteristics of juiciness and flavor [6]. However, lipids are readily oxidized, leading to the formation of toxic and undesirable products [34]. The protein content of meat decreased linearly with increasing licuri cake concentration in the diet; this decrease contributed to the lower slaughter weight [7] of the lambs fed higher concentrations of licuri cake and indicates a possible decrease in the deposition of muscle tissue [35,36].

The pH of the carcass was not affected by including licuri cake in lamb diets. Factors such as pre-slaughter stress and inadequate slaughter procedures are relevant to pH variations, but it is important to note that none of the diets studied had deleterious effects on the pH of the meat. The pH values remained within the range considered essential for the proper establishment of rigor mortis and transformation of muscle into meat [20]. The final pH values at 24 h after slaughtering varied from 5.82 to 5.92, which are within the normal range (up to 6.0) associated with meat of good quality [6,37].

Color parameters are among the most important attributes when consumers choose meat. The decreased muscle mass deposition [7] with greater licuri cake concentrations (24% of total DM) reduced slaughter weight and resulted in less blood flow and decreased concentrations of sarcoplasmic proteins and other pigments in the muscles, which may have changed the redness and chroma color indexes [38]. Thus, reducing carcass weight gain promotes lower muscle fiber development which may have caused a reduction in myoglobin deposition, resulting in less-pigmented meat [37, 39].

Cooking loss and WBSF values were similar among treatments. In this research, all the meats sampled were considered to be tender (2.44 to 1.54 kg*f*/cm^2^) because according to Webb et al. [40] a WBSF up to 3.0 kg*f*/cm^2^ is an acceptable value for tenderness.

### Fatty acid profile

The lipid deposition in meat has been shown to decrease due to licuri cake inclusion in the diet of lambs because the cakes decreased the DMI from the lambs’ diet, and lipids were metabolized and stored in adipose tissues, which affected the meat [8]. The adverse effects of EE on nutrient intake from diets with higher inclusion of licuri cake are amplified by the degree of unsaturation and chain length of the FA component of the vegetable oil from biodiesel by-products such as palm kernel cake, sunflower cake, peanut cake which has been emphasized in previous studies [2,4,7,39].

Values from 14.7 to 23.8 g/kg in the lipid concentration of *longissimus lumborum* are considered normal for a slaughter between 15 and 45 kg. In addition, the amount of lipids in the muscle can be increased by the inclusion of oilseed cakes in the lambs’ diet [39,41,42]. The change in lipid concentration in the meat of the animals may reflect differences in the FA composition of the meat. The inclusion of licuri cake in the diet of lambs increased specific SFAs (C12:0, C14:0, C17:0, and C20:0) and were promoted by a high proportion of ΣSFA in the licuri cake in comparison to other feed ingredients. The lipid content of the meat can alter its lipid profile [6,8,41]. This occurs due to differences in the ratio of phospholipids, which are polar fats, to triacylglycerols, which are neutral storage fats [43,44].

In comparison to findings in the literature [12,34,35,45], the *longissimus lumborum* of our study groups showed a greater quantity of MUFA, mainly palmitoleic (C16: 1) and oleic (C18: 1) acids, followed by the concentration of SFA palmitic acid (C16: 0), myristic acid (C14: 0), and stearic acid (C18: 0) specifically, as well as the latter PUFA, which is represented mainly by linoleic acid (C18: 2). The reduction in the amount of MUFA and an increase in the amount of SFA by the addition of the licuri cake also influenced the ratios of ΣMUFA: ΣSFA and promoted an increase in ΣPUFA: ΣSFA, due to the tendency of increase of PUFA. This tendency of ΣPUFA increase is important because they are located in cell membranes and are precursors of different eicosanoids (prostaglandins, thromboxanes and leukotrienes), which act as messengers of the cell and metabolic regulators and whose specific functions are of particular interest in the study of cardiovascular diseases [46,47,48]. In addition, the benefits of its use are associated with its performance in maintaining the integrity of the cell membrane and its ability to decrease the amount of serum lipids [42,47].

The ratio of Σ*ω-6*:Σ*ω-3* fatty acids, which should be lower than 4.0, has also been used as a criterion for assessing fat quality [47,48]. In the present study, although the values ​​were above the established range, animals fed a diet that contain licuri demonstrate a strong reduction in the ratio of Σ*ω-6*:Σ*ω-3* fatty acids from 13.68 to 5.21.

Due to their positive effects on human health, fatty acids are of particular interest–especially linolenic acid (C18:1) and linoleic acid (C18:2), which are considered essential, in addition to fatty acids with carbon 3 unsaturation, CLA and C20:5 [45,46]. The content of the latter two were not influenced, but the content of C18:3 n-6 and C18:3 n-3 and, were higher in the animals consuming licuri cake. The concentration of CLA in foods varies widely, but the greatest concentrations are generally in foods derived from ruminants [44]. Diet is the primary factor for the enrichment of CLA in lamb products (meat and milk) [47, 48]. Foods of ruminant origin (i.e., meat and milk products) typically have CLA concentrations between 3% and 7% of the total fat, which, as previously discussed, may be significantly affected by diet [1,4]. In this study, the mean CLA concentration was 2.6%, which is below the range indicated by previous authors, most likely due to the reduced fat content of the meat.

The inclusion of licuri cake in the lamb diets did not influence the DFA and h:H ratio, which has an average of 0.58. However, the highest licuri cake inclusion (24% of total DM) promoted an increase in the AI index of the *longissimus lumborum* of the lambs. According to the literature, an h:H ratio of up to 2.0 in meat is also considered satisfactory and does not represent a risk to human health [28,47,48], since its relationship represents the effect of fatty acids on the mechanism of transport of cholesterol by lipoproteins, which influences the onset of cardiovascular diseases [47,48]. The AI assesses the plaque formation capacity of blood vessels. The lower AI value and the more anti-atherogenic FA will be present in the lipids, thereby leading to greater prevention of cardiovascular diseases [46,47]. In this present study, the average AI values observed decreased from 0.60 to 0.79 for diets with 0% to 16% licuri cake inclusion, respectively, which was lower than the average found by Ulbricht and Southgate [9] for lamb meat (1.00).

### Sensory attributes

The effects of licuri cake on the moisture of lamb meat might have led to some significant differences in the sensory attributes reported by the panelists, and it is likely that the water content directly influenced the taste and odor of the meat. According to Muela et al. [49], consumers prefer thawed and fresh meat, and the concerns of consumers regarding unfrozen meat should be reconsidered. Water retention capacity is undoubtedly an important quality factor because it plays a role in how the attributes of meat are affected before and after cooking and because of the influence it has on juiciness during mastication. However, differences were expected because the lipid contents in the *longissimus lumborum* of lambs were lower in the treatments with greater licuri cake concentrations. Although to a lesser extent than water, lipids are important components of meat because they improve desirable sensory characteristics, such as juiciness, flavor, and aroma [50,51].

The average classification scores by the evaluators of characteristics such as aroma and flavor were similar on the hedonic scale (rankings averaged between 6.17 and 7.38), that is, despite presenting a significant difference to these characteristics, no negative feedbacks have recorded in treatments, presenting soft to moderate sympathy by the panelists. However, the meats of animals that received 24% licuri cake inclusion obtained the highest averages. In general, the samples were acceptable to the tasters, with ratings of “liked slightly” to “liked moderately”, which is a positive result commercially because tasters would recommend the meat of these animals for consumption by humans [47,48].

The increase in SFA and PUFA contents of the *longissimus lumborum* of lambs as a result of licuri cake inclusion is another aspect that is relevant to changes in the flavor of the meat [30, 48]. Meat aroma and flavor are directly related to the FA profile present in the meat [42], especially that of PUFAs, which provide better flavor and consequently improve overall acceptance. This is because PUFAs are particularly prone to oxidative degradation and to the production of volatile compounds, which are associated with the development of off flavors in meat. Therefore, the increase in meat flavor rankings with licuri cake supplementation (24% of total DM) is probably related to the greater *n−3* PUFA content of meat, as suggested by the positive correlation between those two variables [6].

## Conclusion

Licuri cake inclusion in the diet of lambs improves sensory attributes of meat and the meat fatty acid profile becomes nutritionally healthier for the human diet because do not affect major FA of meat; however, the growth performance of finishing lambs is reduced.

## Supporting information

S1 File Fatty acids table. (XLSX)
